# How initial policy responses to COVID-19 contributed to shaping dying at home preferences and care provision: key informant perspectives from Canada

**DOI:** 10.1186/s12913-023-10340-x

**Published:** 2023-11-30

**Authors:** Maria Cherba, Laura Funk, Erin Scott, Bora Salman, Andrea Rounce, Corey Mackenzie, Kelli Stajduhar, Carren Dujela, Marian Krawczyk, S. Robin Cohen

**Affiliations:** 1 University of Ottawa, Ottawa, Canada; 2 University of Manitoba, Winnipeg, Canada; 3 University of Victoria, Victoria, Canada; 4 University of Glasgow, Glasgow, Scotland; 5 McGill University, Montréal, Canada

**Keywords:** Canada, COVID-19, End of life care, Palliative care, Public policy, Thematic analysis

## Abstract

**Objectives:**

In response to COVID-19’s first wave, provincial governments rapidly implemented several public health directives, including isolation measures and care facility visitor restrictions, which profoundly affected healthcare delivery at the end of life and dying experiences and perceptions. The objective of this study was to identify implications of early policy changes for dying at home.

**Methods:**

Analysis of interviews with 29 key informants with expertise in the policy and practice context of dying at home and care for those dying at home was conducted as part of a larger mixed-methods study on dying at home in Canada.

**Results:**

Initial pandemic policy responses, especially visitor restrictions and limitations to home care services, shaped dying at home in relation to three themes: (1) increasing preferences and demand for, yet constrained system ability to support dying at home; (2) reinforcing and illuminating systemic reliance on and need for family/friend caregivers and community organizations, while constraining their abilities to help people die at home; and (3) illuminating challenges in developing and implementing policy changes during a pandemic, including equity-related implications.

**Conclusion:**

This study contributes to broader understanding of the multifaceted impacts of COVID-19 policy responses in various areas within Canadian healthcare systems. Implications for healthcare delivery and policy development include (1) recognizing the role of family/friend caregivers and community organizations in end-of-life care, (2) recognizing health inequities at the end of life, and (3) considering possible changes in future end-of-life preferences and public attitudes about dying at home and responsibility for end-of-life care.

**Supplementary Information:**

The online version contains supplementary material available at 10.1186/s12913-023-10340-x.

## Introduction

In March 2020, Canada’s provinces and territories declared states of emergency due to COVID-19 [1]. Governments issued numerous public health directives to contain the spread of the virus. These responses included enforcing strict isolation measures and visitor restrictions across hospitals, nursing homes/long-term care, and retirement and assisted living residences, in order to protect patients and residents (many of whom are older adults), as well as limit exposure for staff and safeguard limited personal protective equipment [2]. Due to these measures, some people found themselves isolated at the end of life with limited control over how and where they wanted to spend their last days or with whom [3]. This situation contradicted “conventional societal perspectives on what it means to die well” [4] and the palliative care principle that families are part of the unit of care [5]. Indeed, the implementation of severe visitor restrictions in care settings across Canada during the first wave of the pandemic (also a common practice in other countries [6, 7]) was widely criticized for its negative impacts on families and care providers. Media coverage highlighted accounts of hospitalized patients and long-term care residents dying alone, in some instances lacking basic necessities (even to the point of dying from dehydration), and of families deciding to care for older or dying family members at home due to fears and uncertainty about visitation [8–14].

These and similar stories may have longer-term implications for end-of-life attitudes, beliefs, and care decisions of the Canadian public. Developing public health directives and providing end-of-life care services are particularly challenging during emergencies such as pandemics, where urgent responses to immediate risks can fundamentally conflict with palliative care philosophy. This is especially the case when such responses involve “restricting visiting policies, abandoning end-of-life care discussions, not adhering to patients’ end-of-life care wishes […and] disallowing patients and families the usual death and bereavement rituals” [15].

Dying at home and accessing palliative services in one’s home or community are key indicators of high-quality care in Canada [16]. However, access to end-of-life care at home remains challenging in the country [17]. In Canada, provincial and territorial governments are primarily responsible for providing most health care services, including home care [18]. Coverage is universal for medically necessary hospital and physician care (under the *Canada Health Act*) although coverage of allied health services (e.g., physiotherapy, psychology) varies among the 13 systems [19]. Long-term care homes (also called nursing homes or residential care homes) in the country can be publicly owned (not-for-profit) or privately owned (for-profit or not-for-profit), and most receive public funds for the provision of services [20, 21]. In end-of-life care, in addition to government health care systems, community and volunteer services (including unpaid family caregivers) play an important role in service delivery [22]. Hospice organizations in Canada are mostly funded by donations (and in part by government) and contribute significantly to end-of-life care provision, along with other volunteer and non-profit organizations that strive to provide care in communities that lack access to formal services [22]. Exploring the issues of dying at home in the context of COVID-19 mitigation measures offers insights for healthcare providers, administrators, and public health policy development. To inform future pandemic and emergency response, a better understanding of the multifaceted impacts of pandemic policies is needed, including “far-reaching and unintended” consequences in various areas and on different groups and organizations [23]. The objective of this study is to describe changing policies and practices during COVID-19’s first wave and trace the implications of these early responses for dying at home in particular.

## Methods

This study is based on individual interviews with 29 key informants (professionals, volunteers, and community advocates with expertise in the policy and practice context related to dying at home and end-of-life care) in three Canadian provinces: British Columbia, Manitoba, and Québec. Interviews were conducted virtually between July 2020 and June 2021. As end-of-life care is a narrow field of policy and practice, participants were recruited through the research team’s professional networks and snowball sampling [24, 25]. Participant profiles are outlined in Table 1. To preserve confidentiality, interview quotes in this article are identified by profession only when it is necessary to understand the quote.

**Table 1:** Interview participants

Province and number of participants	Generalized description (to preserve confidentiality)
National (n = 2)	Policy analyst, non-profit executive.
British Columbia (n = 11)	Government (regional and provincial) and non-profit executives, clinical nurse specialist, palliative care volunteer and patient advocate, compassionate care community members, frontline inner-city service provider.
Manitoba (n = 6)	Government (regional and provincial) and non-profit executives, care coordinators (palliative care, rural older adults), member of a volunteer organization that supports older LGBTQ2 + adults.
Québec (n = 10)	Government executives (regional and provincial), researchers (university and government), social workers, palliative care nurses, home care professionals, palliative care physicians.

Ethics approvals were obtained from the co-authors’ universities and, where applicable, relevant health authorities. All participants provided written informed consent before taking part in the study. Participants did not receive any remuneration. Semi-structured interviews of approximately one hour were conducted in English or French by M.C., L.F., E.S., B.S., and two research assistants (for this paper, French quotes were translated into English). Two participants preferred not to be recorded; they reviewed and approved detailed interviewer notes. Interview questions (Additional File 1) focused on policy development related to care for persons dying at home, public opinion and preferences related to home dying and care responsibilities at the end of life, and how policy and healthcare practice in this regard changed due to the pandemic. The interview questions were developed as part of a larger study on dying at home in Canada [26]. The analysis in this article focuses specifically on the interview data related to the policy responses to COVID-19. This is the first publication presenting the results of the interviews. Recruitment continued until the data allowed for in-depth description of the impacts of policy changes on dying at home, from the perspectives of people in different sectors including government agencies, health and social care, and non-profits. Our goal was to obtain rich data from a variety of participants in various care contexts, to account for the multifaceted implications of the pandemic for end-of-life care at home [27].

We conducted an inductive thematic analysis of interview transcripts [28, 29]. Initial coding (conducted mainly by M.C., with support from L.F., E.S., and B.S.) focused on descriptions of operating policies, implementation and changing practices during the first wave of the pandemic, as well as implications for end-of-life preferences and end-of-life care provision. During the second stage of coding (conducted by M.C. and progressively discussed with L.F., E.S., B.S., and A.R. during a series of data analysis meetings), three larger themes (presented in the results section below) were identified, along with some overarching equity-related implications (for instance, participants referred to how policy responses to COVID-19 revealed or created inequities in access to services for some population groups, and how they may have differentially affected the experiences of different communities). We explored potential differences between provinces by comparing responses from participants from the three included provinces. Ultimately, however, overall policy responses to COVID-19 and challenges related to dying at home were largely similar among participants across provinces. Analysis was further guided by the “problem representation” framework, focused on understanding what “problems” policy changes are addressing and identifying their underlying assumptions [30]. Following this approach, during the analysis we paid attention to how key informants discussed the values and principles behind policy actions. Discursive tensions in this regard were identified, such as the need for family caregivers as “essential service providers” and the need to monitor these care providers through “visitor” policies.

The analysis was reviewed by all co-authors in more advanced stages, during two team meetings to discuss emerging findings. In addition, initial results were presented to the research team and 27 key stakeholders (including some who took part in the interviews, as well as other experts and professionals from eight Canadian provinces and territories) during a virtual workshop in May 2022. The goal of the workshop was to present the research findings to the participants and engage in a deeper discussion about how the findings relate to their work and may inform change. This presentation and discussion validated the main analytic themes, confirmed their relevance to various provinces, and helped us refine analysis through an equity lens as workshop participants reinforced the importance of an equity framework in future policy development and implementation.

## Results

In the sections below, we present three themes describing how initial pandemic policy responses contributed to shaping dying at home preferences and care provision.

### Shifting end-of-life preferences and awareness

Participants commonly identified increased patient and family preferences for dying (and receiving end-of-life care) at home as an implication of the pandemic context and related policy and practice shifts. For instance, participants described situations where families were moving family members out of institutional care or where patients were unwilling to go to hospitals or palliative care units, in part because of visitor restrictions. In this context, an increased desire and demand for palliative care at home amplified long-standing issues around access to home care. This was further exacerbated by COVID-19 policies restricting home care visits. Participants explained how tension between increased desire to die at home and the lack of home care services could lead to difficult experiences and decisions:

[…] deciding to keep people at home instead of putting them in a facility because they want to see loved ones, but having issues with pain management, and other things they couldn’t do because they aren’t in a hospital.

COVID has possibly created less desire to die in the hospital because of the strict visitation policies […] [and] may have increased desire to want to die at home. […] [But] some of the visits from the palliative care program […] are no longer happening. For some it means they can’t die at home and they have to go into hospital because of the level of care they would have had prior to COVID [is now too high to be met].

Other participants described how restrictive pandemic measures had some unexpected positive implications for palliative care services at home. For instance, healthcare teams had to adopt more integrative approaches to home-based specialist services to avoid transfers to hospitals or other institutional care settings. Participants also mentioned enhanced collaboration between home/community care workers and hospital teams, rapid development of training modules and tool kits in palliative care and pain management for healthcare professionals, implementation of telephone or online consultations, and other additional efforts by different healthcare professionals “to build capacity within our system to keep people at home as long as possible”:

[…] home care and other providers that traditionally would not have been looped in or would not have been as needed to get involved in palliative care […] with a little extra support and different providers stepping in, can definitely provide that great model of palliative care services at home rather than […] rely on institutions – especially hospitals.

Participants talked about pandemic restrictions as requiring healthcare teams to think adaptively and creatively to help more dying persons to stay at home, but simultaneously emphasized that “dying at home is really only feasible if you have 24/7 medical or nursing support […] [and family/friend] caregivers who can actually reliably and consistently meet your care needs” at home.

Some participants identified how increased desire for and experiences of dying at home meant that more people were exposed to already-existing “practical realities of dying at home” (including home-based service limitations):

I think dying at home is this sort of idea that people talk about, but it’s not often talked about in terms of practical realities […] in our health care system that for the first time have been made real for some people […] Sorry, we have this much resource and you are not on the list for that. We are not going to ever say it in that way, but there is a bit of that consciousness now that […] there are some constraints and the pandemic has made that evident.

Other participants described how the pandemic more broadly revealed an existing lack of public death literacy, including awareness about end-of-life care, advance care planning, and grief. Low death literacy contributed to challenges having conversations about death and dying, at a time when there was increased need for these conversations:

[…] those who are experiencing grief in a COVID environment where public health restrictions just restrict so many things… restrict your ability to get together as a family, sometimes restricting their access to the person if they were up in the hospital for the last days of life… funeral rituals, all of those things. So we need public education to help people understand […] the grieving process, because very few Canadians understand it.

[…] advance care planning […] as we saw through the pandemic, there’s not a lot of people that have talked about what their wishes were and then families were sort of struggling without those decisions.

In sum, participants’ insights indicated how pandemic-related policy and practice shifts may have contributed to increased desire to die at home, while service-related changes and other restrictions (in addition to existing realities of limited services and low death literacy) often made it more difficult to do so, even as healthcare teams strove to adapt and innovate.

### Making family/friend caregivers and community organizations visible

Participants referred to how the pandemic exacerbated a tension between the essential role of many ‘informal’ care providers and their lack of recognition and support. For instance, they talked about how policy changes were implemented “through a hospital lens”, putting the experience of family caregivers and home care “at the bottom of the list”:

[…] with a very hospital-centric vision, we were worried about having enough beds, about personal protective equipment […] it’s really, really good, but we forgot about the majority of people who are not in the hospital. The majority of people are not in the hospital, they are not in palliative care facilities, they are at home, and we didn’t tend to this.

[…] we completely put [families] on the sideline […] we stop[ped] famil[y] visits, but we authorize[d] the army to go take care of [people in healthcare facilities]. […] we try so much to advocate for the palliative approach, the wholeness, [and] then we put a player who is a stakeholder in it completely on the side.

Early visitor restrictions in institutional care settings both contributed to and revealed gaps in care in those settings, which in turn illuminated how family and friends were assisting dying persons, including instrumental help with activities of daily living and social support. In this regard early restrictions contravened both the palliative care principle of considering the family as part of the unit of care and that of addressing the physical, psychological, social, spiritual, and practical needs of dying persons [5]. One participant talked about the tension, exacerbated by the pandemic, between holistic palliative care philosophy and the biomedical approaches that guide the development of healthcare policy:

[…] the development of palliative home care policies [is] extremely health-oriented […] We are here in [province], and I am thinking elsewhere in Canada, in a very hospital-centred […] a highly medicalized vision of health, when we know that […] dying at home is not just about having access to a doctor […] it’s really critical that we expand palliative care policy to the psychosocial and spiritual aspects […] palliative care is not just about medical management.

Some participants also explained how the provision of quality end-of-life care at home was directly linked to access to family caregivers:

[…] if we could do better in supporting family caregivers, I think we would see an increase in quality end-of-life care at home and people staying at home […] I don’t think our health system can support people and I think it takes a community.

Two participants involved in the development and implementation of visitation rules highlighted tensions between palliative care philosophy, and more specifically having access to family, and the urgency of quickly implementing measures to contain the spread of the virus:

Of course, the whole question of family caregivers, if we could, we would have done it differently. Since we have a system that is deficient in terms of its capacity to provide care, we must understand that family members are the ones who often fill the gaps, they are the ones who will be mobilized to provide care and services that the healthcare system cannot provide.

The first wave, we learned, was very restrictive, it was very difficult. It highlighted […] the dichotomy between infection prevention and control and palliative and end-of-life care, one was against the other, and we had to correct that. […] [F]or the second wave […] we put in place a significant relaxation of restrictions, to allow people, no matter where they are, to have access to their family.

These participants described numerous complaints and critiques from citizens, media, and community organizations in response to restrictive policies. This was especially prominent in the context of long-term care, and some respondents highlighted how “the fact that [family caregivers] were not able to be present when […] their person was dying in long-term care will have some lasting effects”. Stakeholders in some regions also described how their organizations advocated for government to reconsider visitor restrictions. Indeed, subsequent directives after the first wave were adapted in some regions to recognize some family caregivers as “essential care providers” to allow them to access those settings, especially caregivers of persons nearing the end of life. This may have helped mitigate the most immediate impacts on dying at home preferences that were outlined in the previous section. In a broader sense, participants talked about the pandemic as a call to action to advocate provincially and nationally for caregivers and for home care.

As governments were relying on community organizations to provide more care to people dying at home in the context of limited institutional and home care resources, some provinces allocated additional funding to non-profit hospice and palliative care organizations, including those supporting families providing end-of-life care. Nonetheless, participants mentioned that many organizations still faced financial and other pressures: many events and awareness campaigns were cancelled, donations reduced, and organizations had to lay off workers and limit volunteer engagement. For instance, an executive from a national non-profit noted that “donations have dropped about 70% across the country”. Another executive from a provincial charity explained that “with COVID-19, unfortunately we had to stop our in-home visits […] our volunteering visiting programme.” Pressures on non-profit organizations affected home supports and support for home-based family/friend caregivers, and further aggravated limitations in end-of-life supports related to pandemic restrictions. A government executive talked about how these pressures are related to the funding model for end-of-life care:

Non-profit organizations play an important role in the delivery of end-of-life services. Hospices in [province] are 50% funded by the government and 50% by non-profits. And in the current context, when their ability to fundraise is limited, we have increased their funding. The question is, how much can we really rely on our organizations to provide essential services, given that their funding and the availability of the services they provide are not guaranteed.

In the context of pandemic restrictions and staff shortages, participants also emphasized how families assumed more care responsibilities for dying persons, taking on some tasks previously provided by home care workers. Participants talked about some “family caregivers having to take on the role of being a family caregiver when they didn’t expect to” without having access to adequate supports, which in turn affected their wellbeing (for example, experiences of grief and higher psychosocial support needs that were not met). As such, participants reflected on how the pandemic highlighted the question of the distribution of responsibilities for end-of-life care between government, community, and family:

[…] [the burden of] palliative care is really rested on the loved ones and family. When you get cancer, all the treatments [are] covered, you have a cardiac problem, it’s cared for by the health system. When we are at the end of our life, well then, we are in a collective project […] we ask for community and family involvement that’s really important […] and that also allows some savings for the state, but it’s also as though the responsibility for end-of-life care, in our culture, is a responsibility shared amongst the network, the family and the community, the community organizations. I’m not saying that it’s good or that it’s bad, I just mean that the state has a more limited role [in palliative care] than in other sectors.

Participants talked about shared responsibility as a community strength but also as a limitation with regards to the provision of reliable services and support at the end of life, for instance as community-based services are highly dependent on charitable donations and funding, meaning their availability is not guaranteed. In addition, participants described how, by contributing to and revealing reliance on family/friend supports at the end of life, pandemic-related restrictions likewise contributed to and revealed inequities in access to end-of-life care (and thus people’s ability to spend their last weeks of life at home) for different communities:

[…] it engenders inequities, because, depending on your social network, on your family network, on the strength of these networks, or the resources available or not available in your community, that will make your end of life easy or very, very difficult.

In sum, participants emphasized how policy changes around visitations and home care during the pandemic’s first wave both contributed to a greater strain on family/friend caregivers and community organizations while making their role in end-of-life care more visible across various settings, including at home. Participants also reflected on how the pandemic brought to public attention the question of distribution of care responsibilities and related equity implications.

### Challenges in implementing policy changes

Participants frequently spoke of challenges in developing and implementing policy changes given different interpretations of province-wide directives by health authorities and care facilities, depending on their location and available resources. For example, one government executive explained that the government wanted to give some flexibility to institutions, because of regional variation in service capacity. They described how they worked with organizations on a case-by-case basis to authorize more flexible rules where possible, whereas in other settings stricter rules were used:

[When the directive about easing visitor restrictions was distributed], for some institutions, it was difficult to manage; there are some who will take a stand and tighten up [the restrictions] a little more to be able to manage the number of people. […] and [we] authorized things, for example, in more remote regions, with bigger families, if the healthcare organization tells [us] that they can manage it – please do. So, we give some flexibility to institutions.

Such flexibility, which has also been reported in media and in recent analyses [31, 32], helped some dying persons avoid isolation in facility settings, but also made it “really tough and confusing for family members to know when and how or if they can be at the bedside”. As one key informant explained:

There were many [policy] interpretations, variations, applications in different settings […] [At our palliative care association], we spoke with many people who were saying, can you help us, my dad or my mom or X person is at this palliative care home and they say it’s not the end of life yet, but at this other palliative care home, they interpret it more liberally, yes, he is at the end of life, and so they can accept two people, four people to come visit, they can put a small lounge at their disposal, etc. So, the application of government directives varies from one place to another and it can generate tensions.

This participant further explained how these implementation dynamics can create inequities in access to care:

[Our palliative care association] recently wrote a letter to congratulate the government on the relaxation of these rules to be able to let more people go into facilities so that people aren’t dying alone. But at the same time, we said, look, there is the notion of human and financial resources. Smaller facilities don’t always have enough resources to be able to relax [COVID measures] as well as desired.

More generally, participants contextualized the implementation of pandemic policy changes in relation to existing inequities in access to end-of-life care within each province, especially between urban and remote regions. A priority shared by many participants was for bolstering palliative care resources and to develop services to support dying at home adapted to the realities and healthcare models in different regions. For example, those working in remote regions explained that even if access to “brick-and-mortar hospices” and palliative care physicians is limited in their communities, supporting primary care providers in providing palliative care can help keep people in their home community at the end of life.

In sum, this theme builds on participants’ concerns with how the challenges of implementing early pandemic-related policy changes could have implications for dying persons and their families, not only by contributing to confusion (and hence distress), but also through how efforts to provide flexible rules responsive to local situations may have contributed to variations in how such policy changes shaped end-of-life care preferences as well as access to home-based services in different communities.

## Discussion

Our analysis highlighted the manifold implications of pandemic-related policy changes in COVID-19’s first wave for preferences and experiences of dying at home (and in other settings). We presented three themes describing how initial pandemic policy responses contributed to: (1) increasing preferences and demand for dying at home, alongside often-constrained formal service capacity to support this, (2) reinforcing (while illuminating) systemic reliance on and need for family/friend caregivers and community organizations, while constraining their abilities to support dying at home, and (3) revealing the challenges of developing and implementing policy changes during an emergency, including equity implications for healthcare delivery and access to healthcare.

Our analysis shows how pandemic-related policy changes could shape future preferences and decisions related to the end of life. For instance, more people might prefer dying at home. This preference, however, is perhaps experienced less as a ‘choice’ per se, in the context of the very negative media coverage and the persistent care gaps in institutional settings. A recent survey by the National Institute on Aging [33] found that “85% of Canadians of all ages and 96% of Canadians aged 65 years and older […] will do everything they can to avoid moving into a long-term-care home”. Indeed, one of the most notable shifts in public preferences may be around living and dying in long-term residential care, as some of our other analyses suggest [34]. However, dying at home in a pandemic can also mean spending the last days of life without adequate home care support, including pain management, raising questions about whether home death itself may become less idealized as a result [35, 36]. The extent to which shifts in public preferences might be long-lasting is at this point unknown.

During the early pandemic, families providing care at home were asked to do more but lacked support. Increased public visibility of family/friend caregivers in end-of-life care could encourage more families to become more involved in future care provision. However, to the extent that family care provision at home during the pandemic was associated with negative or unsupported experiences, this could also deter family members from further engagement. In addition, those who themselves provided family/friend care in these difficult circumstances may be less likely to personally want to die at home, out of concerns for burdening the family. In this regard, some participants’ reflections on the effect of these early policy changes on families’ quality of life were noteworthy, since palliative care services are meant to address this [5].

Whereas the role of family/friend caregivers and community organizations in home-based end-of-life care was made more visible in the context of the pandemic, initial policy responses heavily centred on acute and hospital care revealed inadequate public supports allocated to home and community services. As highlighted by the study participants, family and home support is essential to quality end-of-life care. The lack of public support to these services, which makes it impossible for some families to care for a dying person at home, was brought to the public’s attention during the pandemic. In this context, further research is needed on the impacts of COVID-19 on public attitudes about dying at home and responsibility for end-of-life care. In particular, future studies could explore these impacts in relation to the pandemic-related changes in health care delivery that were described by some study participants, such as enhanced collaboration between health care providers and using telemedicine to support access to care at home [37].

Given the complex interconnections between levels of government in Canadian healthcare systems, future studies should also consider the implementation work done by agencies, authorities, and organizations that could affect the experiences and end-of-life preferences of people in different communities. We also need to know more about how public health officials and different health organizations could adapt their communications about policy changes in different settings in a way to avoid confusion and misunderstanding. As examples from our study show, unclear and inconsistent province-wide guidance including the delegation of responsibility to specific organizations negatively affected the experiences of some families and care providers, while at the same time allowing for more compassionate decisions to avoid isolation at the end of life when possible. Other studies of families’ and health care provider’s experiences of care for dying persons during the pandemic suggest that flexible application of rules should be allowed, to help families stay together in the final days [38, 39]. In this context, future pandemic planning and policy should consider this “major task” for health care providers to “continually manage the [pandemic-]associated constraints on an organizational and individual level” [40].

### Implications for practice and policy

The results of our analyses can inform responses to future public health emergencies that support equitable services and facilitate communication with the public about issues related to the end of life. There are two main practice implications of this research. The first concerns the (non)recognition of family/friend caregivers in emergency policies. Although most prominent in institutional policies around restrictions, it was evident in other ways for families caring for dying persons. For instance, home care service prioritization protocols were often structured so as to devolve all but ‘medically necessary’ care to family, even during periods when household visitor restrictions meant that a primary caregiver could not access additional supports from other family and friends. Non-recognition of the role of family/friend caregivers has direct implications for reinforcing health inequities. In terms of home care, not only could the situation have actually reduced some people’s ability to die at home (especially persons without access to family caregivers, or families who could not afford private home care services), but given systemic reliance on women for care labour in Canada [41], there were undoubtedly gendered impacts on health. Moreover, lack of access to family caregivers during the pandemic was particularly difficult for people who may experience additional challenges in receiving care they need because of discrimination, language barriers or conditions such as dementia [3, 13, 42–44], especially in severely understaffed congregate care settings. The urgency of considering how policy responses to COVID-19 may have reinforced existing health inequities [45–47] and of not overlooking family/friend care and well-being in future policy decisions appears even greater in light of pre-pandemic research reports that had already stressed the lack of support for family caregivers, even though some Canadian provinces like Manitoba and Québec legally recognize their contribution [48]. More broadly, interviewed stakeholders’ accounts of their organizations advocating for government to reconsider visitor restrictions indicate how the pandemic may have helped spark greater collective mobilization for family caregivers (especially in institutional settings), as well as broader public debate around the distribution of responsibilities for end-of-life care and inequities in access to end-of-life care.

As researchers and decision-makers continue to examine the consequences of the pandemic, we must ask why family/friend care and well-being have tended to be overlooked in health policies and how we can make sure to fully consider family caregivers’ roles and perspectives, and the potential impacts of policy changes on families’ quality of life, in the future. The tension between palliative care and hospital-centred approaches, that was exacerbated during the pandemic, as well as the impact of the policy shifts on palliative care standards, highlight the importance of integrating palliative care principles, such as family as the unit of care and attention to the whole person care [5], in future emergency responses from the outset. Furthermore, our interviews indicate that family/friend caregiver recognition should go hand-in-hand with acknowledging the inequities that reliance on such forms of care may create for people who don’t have access to such supports (for example, people with limited social networks, those living in regions where community organizations are not present, those whose family caregivers cannot afford to leave their employment, etc.). In addition, we can reflect on how framing family/friend caregivers as essential care providers can inadvertently responsibilize them, or narrow our ability to imagine different forms of caring that are equally worthwhile [49].

The second implication concerns governments’ and healthcare providers’ future communications with the public about dying and the end of life, including the need to raise public awareness about these issues. COVID-19 restrictions negatively affected the experiences of many families. Isolation at the end of life, delayed transfers to palliative care, being unable to follow usual dying and grief rituals (potentially contributing to complicated grief), and other repercussions discussed by participants have also been echoed in recent reports (e.g., [37, 50, 51]). In complex ways, these experiences could have lasting implications for public preferences that should be considered in future public health campaigns and patient-provider conversations about end-of-life care planning.

### Limitations

Study limitations include lack of data from the Canadian provinces and territories that were not included in the interviews, although no difference was found amongst the three included provinces and the results of the study were discussed with stakeholders from more jurisdictions during a virtual workshop as part of the research process. Another limitation relates to the fact that, even though much of the study results focus on the experiences of family caregivers, they were not included as participants in the study, which is an important direction for future research on end-of-life care during the pandemic.

## Conclusion

Drawing on the perspectives of a range of participants with expertise in the policy and practice context related to dying at home and end-of-life care, our study contributes to broader understanding of the multifaceted impacts of COVID-19 policy responses in various areas within Canadian healthcare systems. Implications for healthcare delivery and policy development include (1) recognizing the role of family/friend caregivers and community organizations in end-of-life care, (2) recognizing health inequities at the end of life, and (3) considering possible changes in future end-of-life preferences and public attitudes about dying at home and responsibility for end-of-life care.

In the face of ongoing restructuring of health services and expectations for further public health challenges including contagious disease, supporting preferences for location of dying and death requires awareness of the larger social and epidemiological landscape and the need for flexibility and resilience in policy development and service delivery. Moreover, among the broader public, government communications, as well as advance care planning discussions, need to counter idealization of both family responsibility and dying at home so that families can make informed choices. The need to improve access to quality end-of-life care in different settings has been recognized by governments on different levels and expressed in various principles/definition documents. In this regard, our analyses further emphasize that collective public action is needed to ensure that dying persons have access to family in institutional settings and to home-based services. Both are recognized and prioritized as essential human rights, as having broader equity implications, and as aligning with the principles of palliative care philosophy.

### Electronic supplementary material

Below is the link to the electronic supplementary material.

Supplementary Material 1: The file contains the interview questions for this study.

## Data Availability

The datasets generated and analyzed during the current study are not publicly available as per our ethical approvals, to protect the confidentiality of study participants. Corresponding author, Maria Cherba, maria.cherba@uottawa.ca, can be contacted if someone wants to access the data from this study.
