# Supplementary material for: How initial policy responses to COVID-19 contributed to shaping dying at home preferences and care provision: key informant perspectives from Canada
Source: BMC Health Serv Res. 2023 Nov 30;23:1330. doi: 10.1186/s12913-023-10340-x (PMC10691158; doi:10.1186/s12913-023-10340-x)
Supplement: Supplementary file 1 — Supplementary Material 1: The file contains the interview questions for this study. [file 12913_2023_10340_MOESM1_ESM.docx]

Additional File 1

Interview Questions

1. Can you name some of the key policy leaders or groups involved in shaping and implementing policy related to paid and unpaid care for persons dying at home?
2. What shapes the development and funding of policies in this field?
3. Can you speak about how policies are implemented in this field?
4. In what ways have COVID-19 influenced the ability for people to die at home? Have there been any policy or practice changes in your region in this regard?
5. How has COVID-19 has impacted policy considerations and implementation related to paid and unpaid care for persons dying at home?
   1. Probes: benefits/challenges/experiences/interests of rapid policy development in relation to dying at home/caregivers of those dying at home?
6. How effective generally, are the policies and programs that are currently in place, for supporting people who wish to die at home? And their family members?
7. Is there anything we should add to/remove from our list of policies relevant to dying at home in Canada (*Interviewer note - ask participant to* *refer to the Excel file we sent them in advance*)? Other reactions?
8. Do you believe current policies and programs related to dying at home in Canada match well with public opinion? Why/why not?
9. What advice do you have for our team as we look further into Canadian policies relevant to dying at home?
10. What other people or groups would you recommend that we talk to about this?
